# Etymologia: Petri Dish

**DOI:** 10.3201/eid2701.ET2701

**Published:** 2021-01

**Authors:** Monika Mahajan

**Affiliations:** Post Graduate Institute of Medical Education and Research, Chandigarh, India

**Keywords:** etymologia, Petri dish, Petri plate, bacteria, microbiology, Julius Petri, Robert Koch

## Petri Dish [pe′tre ′dish]

The Petri dish is named after the German inventor and bacteriologist Julius Richard *Petri (1852–1921*). In 1887, as an assistant to fellow German physician and pioneering microbiologist Robert Koch (1843–1910), Petri published a paper titled “A minor modification of the plating technique of Koch.” This seemingly modest improvement (a slightly larger glass lid), Petri explained, reduced contamination from airborne germs in comparison with Koch’s bell jar.

Similar alterations had been suggested earlier by Slavonian researcher Emanuel Klein (1844–1925), who was working in England and described a nearly identical dish in his 1885 book *Micro-organisms*. An 1886 research paper published by Percy Frankland (1858–1946) in the *Proceedings of the Royal Society* portrayed a comparable shallow, circular, and covered dish. Available historical complications accord credit of discovery of the Petri dish to other bacteriologists (Figure).

**Figure:**
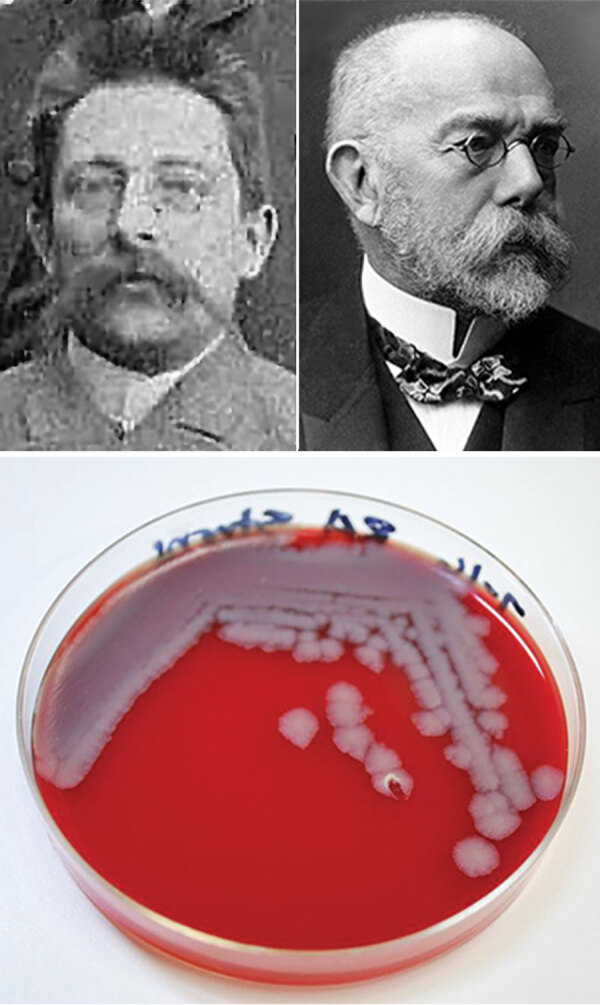
Top left: Julius Richard Petri, inventor of the Petri dish, »1888. Unknown photographer, from file Gruppenaufnahme von Bakteriologischen Kursen im RKI um 1888-A.jpg, Public Domain, https://commons.wikimedia.org/w/index.php?curid=31684326. Top right: Robert Koch. Unknown photographer, from the National Institutes of Health, US Department of Health and Human Services. Bottom: Petri dish showing *Bacillus anthracis* bacterial colonies grown on sheep’s blood agar for 24 hours. Photograph, Centers for Disease Control and Prevention/Megan Mathias and J. Todd Parker, 2009
